# Supplementary material for: Evaluation of nutrient content of different harvest stages in switchgrass (Panicum virgatum L.) cultivars
Source: PeerJ. 2024 Nov 26;12:e18570. doi: 10.7717/peerj.18570 (PMC11606328; doi:10.7717/peerj.18570)
Supplement: Supplemental Information 1 [file peerj-12-18570-s001.docx]

**Supplementary Table 1 Binary and triple interaction values of P, K and Ca properties**

| Cultivars | Year x Cultivar x Harvest stages (HS) | | | | | |
| --- | --- | --- | --- | --- | --- | --- |
|  | 2019 | | | 2020 | | |
|  | HS1 | HS2 | HS3 | HS1 | HS2 | HS3 |
|  | P | | | | | |
| Kanlow | 0.198 | 0.195 | 0.195 | 0.205 | 0.203 | 0.198 |
| Shelter | 0.235 | 0.215 | 0.203 | 0.220 | 0.235 | 0.203 |
| Shawnee | 0.233 | 0.208 | 0.208 | 0.230 | 0225 | 0.200 |
| BoMaster | 0.230 | 0.210 | 0.198 | 0.215 | 0.210 | 0.198 |
| Alamo | 0.213 | 0.198 | 0.203 | 0.205 | 0.203 | 0.190 |
| Trailblazer | 0.235 | 0.223 | 0.213 | 0.230 | 0.198 | 0.188 |
| Cave in Rock | 0.205 | 0.195 | 0.198 | 0.225 | 0.223 | 0.198 |
| Long Island | 0.250 | 0.228 | 0.213 | 0.225 | 0.133 | 0.193 |
|  | Year x Harvest stages | | | | | |
| Average | **0.225 A** | **0.209 CD** | **0.203 D** | **0.220 AB** | **0.216 BC** | **0.196 E** |
|  | K | | | | | |
| Kanlow | 0.393 | 0.500 | 0.523 | 1.055 | 0.863 | 0.788 |
| Shelter | 1.035 | 0.848 | 0.450 | 1.482 | 1.275 | 0.853 |
| Shawnee | 0.890 | 0.665 | 0.508 | 1.550 | 1.228 | 0.925 |
| BoMaster | 1.003 | 0.643 | 0.470 | 1.163 | 1.053 | 0.778 |
| Alamo | 0.580 | 0.300 | 0.675 | 0.938 | 0.923 | 0.728 |
| Trailblazer | 0.978 | 0.617 | 0.585 | 1.317 | 0.930 | 0.733 |
| Cave in Rock | 0.588 | 0.460 | 0.428 | 1.208 | 1.163 | 0.865 |
| Long Island | 1.150 | 0.858 | 0.740 | 1.353 | 1.298 | 0.895 |
|  | Year x Harvest stages | | | | | |
| Average | **0.827 C** | **0.611 D** | **0.547 D** | **1.233 A** | **1.091 B** | **0.820 C** |
|  | Ca | | | | | |
| Kanlow | 0.500 a-g | 0.475 a-g | 0.443 c-g | 0.530 a-g | 0.518 a-g | 0.538 a-g |
| Shelter | 0.498 a-g | 0.420 efg | 0.548 a-g | 0.465 a-g | 0.530 a-g | 0.593 ab |
| Shawnee | 0.500 a-g | 0.435 d-g | 0.495 a-g | 0.455 b-g | 0.530 a-g | 0.608 a |
| BoMaster | 0.443 c-g | 0.488 a-g | 0.483 a-g | 0.560 a-e | 0.538 a-g | 0.550 a-g |
| Alamo | 0.475 a-g | 0.513 a-g | 0.480 a-g | 0.553 a-g | 0.500 a-g | 0.575 a-d |
| Trailblazer | 0.490 a-g | 0.468 a-g | 0.538 a-g | 0.488 a-g | 0.515 a-g | 0.528 a-g |
| Cave in Rock | 0.405 g | 0.518 a-g | 0.513 a-g | 0.408 fg | 0.515 a-g | 0.550 a-g |
| Long Island | 0.605 a | 0.578 a-d | 0.555 a-f | 0.550 a-g | 0.585 abc | 0.613 a |
|  | Year x Harvest stages | | | | | |
| Average | **0.489 C** | **0.487 C** | **0.507 BC** | **0.501 BC** | **0.529 B** | **0.569 A** |

HS1:Pre-flowering, HS2: 50% Flowering, HS3: Full flowering
