# Supplementary material for: Evaluation of nutrient content of different harvest stages in switchgrass (Panicum virgatum L.) cultivars
Source: PeerJ. 2024 Nov 26;12:e18570. doi: 10.7717/peerj.18570 (PMC11606328; doi:10.7717/peerj.18570)
Supplement: Supplemental Information 2 [file peerj-12-18570-s002.docx]

**Supplementary Table 2 Binary and triple interaction values of Mg, Ca/P and K/(Mg+Ca) properties**

| Cultivars | Year x Cultivar x Harvest stages (HS) | | | | | |
| --- | --- | --- | --- | --- | --- | --- |
|  | 2019 | | | 2020 | | |
|  | HS1 | HS2 | HS3 | HS1 | HS2 | HS3 |
|  | Mg | | | | | |
| Kanlow | 0.173 | 0.178 | 0.155 | 0.163 | 0.175 | 0.170 |
| Shelter | 0.160 | 0.163 | 0.173 | 0.173 | 0.190 | 0.190 |
| Shawnee | 0.170 | 0.173 | 0.175 | 0.180 | 0.195 | 0.203 |
| BoMaster | 0.158 | 0.168 | 0.165 | 0.180 | 0.188 | 0.190 |
| Alamo | 0.180 | 0.190 | 0.183 | 0.203 | 0.200 | 0.205 |
| Trailblazer | 0.173 | 0.173 | 0.180 | 0.180 | 0.180 | 0.180 |
| Cave in Rock | 0.168 | 0.165 | 0.168 | 0.173 | 0.185 | 0.195 |
| Long Island | 0.190 | 0.185 | 0.185 | 0.220 | 0.212 | 0.215 |
|  | Year x Harvest stages | | | | | |
| Average | **0.171** | **0.175** | **0.173** | **0.184** | **0.191** | **0.193** |
|  | Ca/P | | | | | |
| Kanlow | 2.534 a-h | 2.440 a-h | 2.266 b-h | 2.589 a-h | 2.562 a-h | 2.732 a-f |
| Shelter | 2.119 d-h | 1.954 fgh | 2.710 a-g | 2.060 d-h | 2.263 b-h | 2.940 abc |
| Shawnee | 2.149 c-h | 2.105 d-h | 2.392 a-h | 1.980 fgh | 2.361 b-h | 3.039 ab |
| BoMaster | 1.921 gh | 2.329 b-h | 2.452 a-h | 2.627 a-h | 2.573 a-h | 2.794 a-e |
| Alamo | 2.263 b-h | 2.603 a-h | 2.380 a-h | 2.695 a-g | 2.491 a-h | 3.030 ab |
| Trailblazer | 2.093 d-h | 2.103 d-h | 2.531 a-h | 2.130 d-h | 2.607 a-h | 2.808 a-d |
| Cave in Rock | 1.987 e-h | 2.651 a-g | 2.625 a-h | 1.823 h | 2.310 b-h | 2.790 a-e |
| Long Island | 2.421 a-h | 2.546 a-h | 2.619 a-h | 2.446 a-h | 2.529 a-h | 3.184 a |
|  | Year x Harvest stages | | | | | |
| Average | **2.182 D** | **2.341 BCD** | **2.497 B** | **2.294 CD** | **2.462 BC** | **2.915 A** |
|  | K/(Mg+Ca) | | | | | |
| Kanlow | 1.755 abc | 1.391 abc | 1.242 bc | 0.661 c | 0.820 c | 0.996 bc |
| Shelter | 0.647 c | 0.718 c | 1.608 abc | 0.445 c | 0.594 c | 0.943 bc |
| Shawnee | 0.758 c | 0.939 bc | 1.336 bc | 0.472 c | 0.567 c | 0.884 bc |
| BoMaster | 0.610 c | 1.041 bc | 1.430 abc | 0.654 c | 0.692 c | 0.943 bc |
| Alamo | 1.329 bc | 2.370 ab | 0.989 bc | 0.814 c | 0.762 c | 1.135 bc |
| Trailblazer | 0.710 c | 1.236 bc | 1.654 abc | 0.519 c | 0.771 c | 0.980 bc |
| Cave in Rock | 1.017 bc | 1.531 abc | 2.886 a | 0.495 c | 0.606 c | 0.878 bc |
| Long Island | 0.703 c | 0.927 bc | 1.101 bc | 0.570 c | 0.617 c | 0.924 bc |
|  | Year x Harvest stages | | | | | |
| Average | **0.941 BCD** | **1.270 AB** | **1.531 A** | **0.579 D** | **0.680 CD** | **0.963 BC** |

HS1:Pre-flowering, HS2: 50% Flowering, HS3: Full flowering
