# Supplementary material for: Evaluation of nutrient content of different harvest stages in switchgrass (Panicum virgatum L.) cultivars
Source: PeerJ. 2024 Nov 26;12:e18570. doi: 10.7717/peerj.18570 (PMC11606328; doi:10.7717/peerj.18570)
Supplement: Supplemental Information 3 [file peerj-12-18570-s003.docx]

**Supplementary Table 3 Binary interaction value of P, K and Ca properties**

| Cultivars | Year x Cultivar | | Cultivar x Harvest stages (HS) | | |
| --- | --- | --- | --- | --- | --- |
|  |  |  |  |  |  |
|  | 2019 | 2020 | HS1 | HS2 | HS3 |
|  | P | | | | |
| Kanlow | 0.196 d | 0.203 cd | 0.201 ef | 0.199 ef | 0.196 f |
| Shelter | 0.218 a-c | 0.222 ab | 0.231 ab | 0.225 a-c | 0.203 ef |
| Shawnee | 0.216 a-c | 0.218 a-c | 0.231 ab | 0.216 b-e | 0.204 d-f |
| BoMaster | 0.213 a-d | 0.208 b-d | 0.223 a-d | 0.210 c-f | 0.198 ef |
| Alamo | 0.204 b-d | 0.199 cd | 0.209 c-f | 0.200 ef | 0.196 f |
| Trailblazer | 0.223 ab | 0.205 b-d | 0.233 ab | 0.210 c-f | 0.200 ef |
| Cave in Rock | 0.199 cd | 0.215 a-d | 0.215 b-f | 0.209 c-f | 0.198 ef |
| Long Island | 0.230 a | 0.217 a-c | 0.238 a | 0.230 ab | 0.203 ef |
|  | K | | | | |
| Kanlow | 0.472 f | 0.902 bcd | 0.724 efg | 0.681 efg | 0.655 fg |
| Shelter | 0.778 cd | 1.203 a | 1.258 a | 1.061 a-d | 0.651 fg |
| Shawnee | 0.688 def | 1.168 a | 1.120 abc | 0.946 b-e | 0.716 efg |
| BoMaster | 0.705 def | 0.998 abc | 1.083 abcd | 0.848 c-g | 0.624 fg |
| Alamo | 0.518 ef | 0.863 bcd | 0.759 efg | 0.611 g | 0.701 efg |
| Trailblazer | 0.727 de | 0.993 abc | 1.148 ab | 0.774 efg | 0.659 fg |
| Cave in Rock | 0.492 ef | 1.078 ab | 0.898 b-f | 0.811 d-g | 0.646 fg |
| Long Island | 0.916 bcd | 1.182 a | 1.251 a | 1.078 a-d | 0.818 d-g |
|  | Ca | | | | |
| Kanlow | 0.473 c | 0.528 abc | 0.515 abc | 0.496 a-d | 0.490 a-d |
| Shelter | 0.488 bc | 0.529 abc | 0.481 cd | 0.475 cd | 0.570 abc |
| Shawnee | 0.477 bc | 0.531 abc | 0.478 cd | 0.483 bcd | 0.551 abc |
| BoMaster | 0.471 c | 0.549 ab | 0.501 a-d | 0.513 abc | 0.516 abc |
| Alamo | 0.489 bc | 0.543 abc | 0.514 abc | 0.506 abc | 0.528 abc |
| Trailblazer | 0.498 bc | 0.510 abc | 0.489 a-d | 0.491 a-d | 0.533 abc |
| Cave in Rock | 0.478 bc | 0.491 bc | 0.406 d | 0.516 abc | 0.531 abc |
| Long Island | 0.579 a | 0.583 a | 0.578 ab | 0.581 a | 0.584 a |

HS1:Pre-flowering, HS2: 50% Flowering, HS3: Full flowering
