# Supplementary material for: Evaluation of nutrient content of different harvest stages in switchgrass (Panicum virgatum L.) cultivars
Source: PeerJ. 2024 Nov 26;12:e18570. doi: 10.7717/peerj.18570 (PMC11606328; doi:10.7717/peerj.18570)
Supplement: Supplemental Information 4 [file peerj-12-18570-s004.docx]

**Supplementary Table 4 Binary interaction value of Mg, Ca/P and K/(Mg+Ca) properties**

| Cultivars | Year x Cultivar | | Cultivar x Harvest stages (HS) | | |
| --- | --- | --- | --- | --- | --- |
|  |  |  |  |  |  |
|  | 2019 | 2020 | HS1 | HS2 | HS3 |
|  | Mg | | | | |
| Kanlow | 0.168 d-f | 0.169 d-f | 0.168 | 0.176 | 0.163 |
| Shelter | 0.165 ef | 0.184 b-e | 0.166 | 0.176 | 0.181 |
| Shawnee | 0.173 c-f | 0.193 bc | 0.175 | 0.184 | 0.189 |
| BoMaster | 0.163 f | 0.186 bcd | 0.169 | 0.178 | 0.178 |
| Alamo | 0.184 b-e | 0.203 ab | 0.191 | 0.195 | 0.194 |
| Trailblazer | 0.175 c-f | 0.180 c-f | 0.176 | 0.176 | 0.180 |
| Cave in Rock | 0.167 d-f | 0.184 b-e | 0.170 | 0.175 | 0.181 |
| Long Island | 0.187 bcd | 0.216 a | 0.205 | 0.199 | 0.200 |
|  | Ca/P | | | | |
| Kanlow | 2.413 a-d | 2.628 a-d | 2.561 a-e | 2.501 a-e | 2.499 a-e |
| Shelter | 2.261 cd | 2.421 a-d | 2.089 ef | 2.108 def | 2.825 ab |
| Shawnee | 2.215 d | 2.460 a-d | 2.064 ef | 2.233 c-f | 2.716 abc |
| BoMaster | 2.234 d | 2.665 abc | 2.274 c-f | 2.451 a-e | 2.623 a-d |
| Alamo | 2.406 a-d | 2.739 a | 2.465 a-e | 2.547 a-e | 2.705 abc |
| Trailblazer | 2.242 cd | 2.515 a-d | 2.112 def | 2.355 b-f | 2.669 abc |
| Cave in Rock | 2.421 a-d | 2.307 bcd | 1.905 f | 2.480 a-e | 2.707 abc |
| Long Island | 2.529 a-d | 2.720 ab | 2.434 a-e | 2.538 a-e | 2.901 a |
|  | K/(Mg+Ca) | | | | |
| Kanlow | 1.462 abc | 0.826 bc | 1.208 abc | 1.106 abc | 1.119 abc |
| Shelter | 0.991 abc | 0.652 c | 0.546 c | 0.643 bc | 1.275 abc |
| Shawnee | 1.011 abc | 0.650 c | 0.615 bc | 0.767 bc | 1.110 abc |
| BoMaster | 1.027 abc | 0.772 bc | 0.632 bc | 0.870 bc | 1.197 abc |
| Alamo | 1.562 ab | 0.904 bc | 1.071 abc | 1.566 ab | 1.062 abc |
| Trailblazer | 1.200 abc | 0.757 bc | 0.615 bc | 1.003 abc | 1.317 abc |
| Cave in Rock | 1.811 a | 0.660 c | 0.765 bc | 1.069 abc | 1.881 a |
| Long Island | 0.910 bc | 0.704 bc | 0.636 bc | 0.772 bc | 1.013 abc |

HS1:Pre-flowering, HS2: 50% Flowering, HS3: Full flowering
